# Transferable Semiempirical Models for Judd–Ofelt Parameters and Multiphonon Relaxation in Eu^3+^ Complexes: Toward an Experiment‐Free Luminescence Design

**DOI:** 10.1002/jcc.70438

**Published:** 2026-06-14

**Authors:** Claudio M. B. Neto, Gabriel S. Santos, Leonardo A. de Souza, Lippy F. Marques, José D. L. Dutra, Ricardo O. Freire

**Affiliations:** ^1^ Pople Computacional Chemistry Laboratory, Department of Chemistry Federal University of Sergipe São Cristóvão Sergipe Brazil; ^2^ Núcleo de Estudos Em Química Inorgânica Teórica (NEQuIT), Instituto de Química, Universidade do Estado do Rio de Janeiro (UERJ), Campus Maracanã Rio de Janeiro Rio de Janeiro Brazil; ^3^ Laboratório de Química de Coordenação e Espectroscopia de Lantanídeos (LQCEL) Instituto de Química, Universidade do Estado do Rio de Janeiro Rio de Janeiro Rio de Janeiro Brazil

**Keywords:** computational design, europium(III) complexes, Judd–Ofelt theory, lanthanide photophysics, quantum yield prediction

## Abstract

The rational design of lanthanide‐based luminescent systems using purely theoretical approaches remains challenging due to the reliance of existing models on experimental data. Herein, we propose a theoretical protocol for the design of new Eu^3+^ based luminescent systems, starting from a previously synthesized and experimentally characterized complex. The approach is based on two semiempirical models: (i) a combination of the *QDC* and BOM models for the calculation of Judd–Ofelt intensity parameters (Ω_2_ and Ω_4_) and (ii) a model based on the van Dijk–Schuurmans equation and Fermi's golden rule to estimate nonradiative emission rates (*A*_NR_) from radiative rates (*A*_R_). The model for calculating the Judd–Ofelt intensity parameters was parameterized using the [Eu_2_(Ibf)_6_(bpy)_2_] complex as a reference and validated with the analogous complexes [Eu_2_(Ibf)_6_(4,4′‐dmbpy)_2_] and [Eu_2_(Ibf)_6_(5,5′‐dmbpy)_2_]. In contrast, due to its mathematical structure, the model for calculating A_NR_ required the inclusion of all three systems in the parameterization set. The models reproduce the intensity parameters with average errors below 4% and the nonradiative rate with errors below 0.2%. The protocol was applied to eight new complexes obtained by substituting the bipyridine methyl group with NH_2_, NO_2_, OCH_3_, and OH at the 4,4′‐and 5,5′‐positions. The theoretical predicted luminescent properties indicate that methoxy‐substituted systems are predicted to exhibit superior performance, with predicted quantum yields of ~93.8%. This protocol provides a computationally efficient strategy for the rational design of Eu^3+^ based luminescent materials.

## 1 Introduction

Lanthanide luminescent systems have found widespread and impactful applications across various fields. In lighting technology, the incorporation of lanthanide ions as phosphors in LED lights has been widely explored to enhance brightness and color rendering [1]. The use of lanthanide‐based luminescent probes in bioimaging has significantly advanced molecular imaging and diagnostics [2]. Furthermore, the utilization of lanthanide luminescent systems in optical devices, such as lasers and amplifiers, has been extensively studied [3, 4, 5]. The applications of lanthanide luminescent systems in environmental sensing, quantum computing, and other fields have been explored in various research articles [6, 7]. These references highlight the broad range of applications and the significant impact of lanthanide luminescent systems in advancing various scientific and technological domains.

The use of theoretical tools for the study of lanthanide‐based luminescent systems has increased significantly over the past two decades. The development of theoretical methods that combine low computational cost and adequate accuracy [8, 9] has contributed to this trend. However, the main reason for the widespread use of these tools has been the development of user‐friendly software designed for studying systems containing lanthanide ions [10, 11, 12]. These tools have been primarily applied to explain phenomena observed in the laboratory that cannot be elucidated through experimental techniques [13, 14, 15]. There are also reports in the literature of using these tools and software to assist in the structural elucidation of synthesized systems, particularly when crystallographic measurements of the system are not feasible [16]. However, the most relevant application undoubtedly lies in using these tools to design highly luminescent systems [17]. Due to the limitations described below, this type of study has been rarely conducted, and when performed, it often involves the application of approximations at certain stages of the theoretical protocol [18].

The theoretical protocol follows the following basic steps: (i) three‐dimensional assembly of structures; (ii) optimization of the ground state geometry of the systems and, if necessary, conformational analysis; (iii) calculation of the energies of the excited singlet and triplet states; and (iv) calculation of luminescent properties, culminating in the calculation of the quantum yield of emission for the systems [19]. The LUMPAC program [10] was developed by our group with the ultimate goal of enabling the design of lanthanide‐based luminescent systems. Currently, we are working to facilitate the design of systems based on Eu^3+^, as it is the most studied lanthanide by the scientific community. In LUMPAC, the step related to the calculation of luminescent properties is subdivided into the following stages: (i) calculation of the Judd–Ofelt [20, 21] intensity parameters based on the ground state geometry; (ii) calculation of energy transfer rates (*W*_ET_) and back‐transfer rates (*W*_BT_) [22]; (iii) calculation of radiative emission rates (*A*_R_) and nonradiative rates (A_NR_) [19]; and (iv) calculation of the quantum efficiency (*η*) and, finally, the quantum yield (*Φ*) [23].

The problem with applying this protocol for the design of luminescent systems is that the calculation steps for the intensity parameters and nonradiative emission rate require the experimental emission spectrum and the ^5^D_0_ → ^7^F_2_ lifetime of the system. This makes it impractical to apply the protocol for the purpose of designing novel systems. Our group has been working diligently for over a decade to develop general methods that are independent of experimental data for calculating the intensity parameters and nonradiative decay rate. However, this is not a simple task. For example, in literature, there are few studies that aim to develop analytical equations for calculating the nonradiative decay rate. In 1983 and 1984, van Dijk and Schuurmans published two works attempting to obtain analytical equations correlating the radiative and nonradiative emission rates, as this would enable the theoretical calculation of the nonradiative decay rate, given that the radiative decay rate can be calculated theoretically from the intensity parameters [24, 25]. According to the authors, this is a long‐standing problem, and in their work, they did not fully resolve the issue.

Since the development of these general theoretical models has been a challenge, in this work, we are presenting a proposed theoretical protocol that allows for the design of luminescent systems based on a previously synthesized system. The idea is to choose a system with its luminescent properties already studied and experimentally characterized. Modifications are made to the outer organic part of the ligands while preserving the coordination polyhedron's geometry as much as possible. Based on the experimental data of the precursor system, parameters in the equations proposed in this work are adjusted to calculate the intensity parameters and the nonradiative decay rate. This will enable the purely theoretical calculation of these properties for the proposed new systems and, ultimately, the calculation of the quantum yield.

## 2 Theoretical Model for Judd–Ofelt Intensity Parameters Calculation

By combining the *QDC* uniqueness model [26] and the BOM model [27], a novel semiempirical approach was established to bypass the empirical dependence of intensity parameters, allowing for the calculation of the Judd–Ofelt parameters from a purely theoretical standpoint. The theoretical intensity parameters, $\Omega_{\lambda }^{\text{calc}}$ (*λ* = 2, 4, and 6), are calculated using the Judd–Ofelt theory [20, 21] which is mathematically described in Equation (1). These parameters aim to describe the interaction between the lanthanide ion and the coordinated ligands.

(1) $$\Omega_{\lambda }^{\text{calc}}=\left(2\lambda +1\right)\sum_{t=\lambda -1}^{\lambda +1\;\left(\mathrm{odd}\right)}\sum_{p=-t}^{t\;}\frac{{\left|B_{\mathrm{\lambda tp}}\right|}^{2}}{2t+1}$$

In Equation (1), the $B_{\mathrm{\lambda tp}}$ parameters are calculated using Equation (2), where the first term, $B_{\mathrm{\lambda tp}}^{ed}$, represents the forced electric dipole (*ed*) contribution (Equation 3), and the second term, $B_{\mathrm{\lambda tp}}^{\mathrm{dc}}$, originates from the dynamic coupling (*dc*) mechanism (Equation 4).

(2) $$B_{\mathrm{\lambda tp}}=B_{\mathrm{\lambda tp}}^{ed}+B_{\mathrm{\lambda tp}}^{\mathrm{dc}}$$

(3) $$B_{\mathrm{\lambda tp}}^{ed}=\frac{2}{\Delta E}\left\langle r^{t+1}\right\rangle \theta \left(t,\lambda \right)\gamma_{p}^{t}$$

(4)

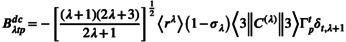




The radial integral values for Eu^3+^ are given as follows: $\left\langle r^{2}\right\rangle$ = 0.9175 a.u., $\left\langle r^{4}\right\rangle$ = 2.0200 a.u., $\left\langle r^{6}\right\rangle$ = 9.0390 a.u., and $\left\langle r^{8}\right\rangle$ = 110.0323 a.u [28]. The ΔE constant indicates the energy difference between the barycenter of the ground state and that of the first excited‐state configuration with opposite parity. The $\theta \left(t,\lambda \right)$ terms in Equation (3) are numerical factors specific to each lanthanide ion, estimated from Hartree–Fock radial integrals [29], with the following values for Eu^3+^: $\theta \left(1,2\right)$ = −0.170, $\theta \left(3,2\right)$ = 0.345, $\theta \left(3,4\right)$ = 0.180, $\theta \left(5,4\right)$ = −0.240, $\theta \left(5,6\right)$ = −0.240, and $\theta \left(7,6\right)$ = 0.240. In Equation (4), the $\left(1-\sigma_{\lambda }\right)$ term accounts for the shielding of the 4f orbitals by the outer orbitals of the lanthanide ion. The $\left\langle 3|\left|C^{\left(\lambda \right)}\right||3\right\rangle$ operator designates the reduced matrix element of a tensor operator of rank *λ* (*λ =* 2, 4, and 6), and $\delta_{t,\lambda +1}$ stands for the Kronecker delta.

The $\gamma_{p}^{t}$ (*t* = 1, 3, 5, and 7) parameter, present in Equation (3), are the so‐called odd‐rank ligand field parameters. These involve a summation over the surrounding atoms and are given by Equation (5), where the *j* index runs over all ligating atoms, and $\rho_{j}$ and $\beta_{j}$ represent corrections introduced by the bond overlap model (BOM) [27] to the crystal field parameters of the electrostatic point charge model (PCEM).

(5) $$\gamma_{p}^{t}={\left(\frac{4\pi }{2t+1}\right)}^{\frac{1}{2}}e^{2}\sum_{j}\rho_{j}{\left(2\beta_{j}\right)}^{t+1}\frac{g_{j}}{R_{j}^{t+1}}Y_{p}^{t*}\left(\theta_{j},\phi_{j}\right)$$

The $\Gamma_{p}^{t}$ (*t* = 1, 3, 5, and 7) parameters, which appear in Equation (4), also depend on the geometry of the coordination polyhedron surrounding the lanthanide ion and are defined by Equation (6).

(6) $$\Gamma_{p}^{t}={\left(\frac{4\pi }{2t+1}\right)}^{\frac{1}{2}}\sum_{j}\frac{\left[{\left(2\beta_{j}\right)}^{t+1}\alpha_{j}^{\mathrm{OP}}+\alpha \prime_{j}\right]}{R_{j}^{t+1}}Y_{p}^{t*}\left(\theta_{j},\phi_{j}\right)$$

As can be seen in Equation (6), according to the BOM model, the effective polarizability is divided into the polarizability of the overlap region ($\alpha_{j}^{\mathrm{OP}}$) and the intrinsic polarizability ($\alpha \prime_{j}$).

The charge factors ($g_{j}$), which appear in Equation (5), and $\alpha_{j}^{\mathrm{OP}}$ are calculated using the analytical equations proposed by Carlos and co‐workers [30]:

(7) $$g_{j}=R_{j}\sqrt{\frac{k_{j}}{{\Delta E}_{j}}}$$

(8) $${\alpha_{j}}^{OP}=\frac{e^{2}R_{j}^{2}\rho_{j}^{2}}{2\Delta E_{j}}$$

where *R*_*j*_ is the lanthanide *j*th ligand atom distance, *k* is the bond force constant, ${\Delta E}_{j}$ is the first excitation energy associated with the chemical bond, which can be considered as the energy difference between the ground and excited states for a given chemical bond, *e* is the electron charge. The overlap integral between the lanthanide ion and the ligand atom given by Equation (9).

(9) $$\rho_{j}=\rho_{0}{\left(\frac{R_{0}}{R_{j}}\right)}^{3.5}$$

where *ρ*_0_ is a constant with a value of 0.05, and *R*_0_ is the shortest distance involving the ligand atoms and the central lanthanide.

For the calculation of ${\Delta E}_{j}$ term, which appears in Equations (7) and (8), Carlos and co‐workers [30] proposed an approximation described by Equation (10):

(10) $$\Delta E_{j}=\varepsilon e^{n\left(\frac{R_{0}}{R_{j}}\right)}$$

where the *j* index is related to each lanthanide‐ligand atom bond, *ε* represents the energy difference between the 4f orbitals and the valence orbitals of the ligand atom in question, *n* is an adjustable parameter, and *R*_*0*_ is the smallest of the *R*_*j*_ distances. It is important to emphasize that for each type of atom bonded to the lanthanide ion (O, N, etc.), there is a different value of *ε*.

Although the polarizability of the overlap region ($\alpha_{j}^{\mathrm{OP}}$) is estimated using Equation (8), there are no equations in the BOM model for calculating the intrinsic polarizability ($\alpha \prime_{j}$). To address this limitation, we proposed calculating it through a linear transformation postulated by the *QDC* model, involving the adjustable parameters *D* and *C* along with the electrophilic superdelocalizability, as shown in Equation (11).

(11) $$\alpha \prime_{j}=SE_{j}\;D+C$$

where the *D* and *C* parameters remain identical for all *j* ligating atoms within a given complex. Beyond parameterizing *D* and *C* to establish a semiempirical framework for the purely theoretical calculation of $\Omega_{\lambda }^{\text{calc}}$, the *ρ*_0_ term in Equation (9) alongside the *n* and *ε* quantities in Equation (10), were also treated as adjustable model parameters. Consequently, our proposed semiempirical model builds upon the equations and comprises the parameter set shown in Table 1.

**TABLE 1:** Description of the semiempirical model parameters used for the purely theoretical calculation of the Judd–Ofelt intensity parameters in Eu^3+^ complexes.

Parameter	Description
^*ρ*^_0_ (O)	BOM overlap factor for the Eu^3+^–O bond
^*ρ*^_0_ (N)	BOM overlap factor for the Eu^3+^–N bond
*n* (O)	Exponential factor in the first excitation energy approximation for the Eu^3+^–O bond
*n* (N)	Exponential factor in the first excitation energy approximation for the Eu^3+^–N bond
*ε* (O)	Pre‐exponential factor in the first excitation energy approximation for the Eu^3+^–O bond
*ε* (N)	Pre‐exponential factor in the first excitation energy approximation for the Eu^3+^–N bond
*D*	Slope of the *QDC* linear transformation for calculating intrinsic polarizability
*C*	Intercept of the *QDC* linear transformation for calculating intrinsic polarizability

### 2.1 Parameterization

Based on the Judd–Ofelt model equations, which exhibit strong dependence on the coordination polyhedron geometry of the compound, we postulate that parametrizing our model for a given compound allows its application to compounds that do not present significant modifications in their coordination polyhedron geometry. Therefore, we selected a series of analogous compounds previously synthesized and characterized by our group.

**FIGURE 1:**
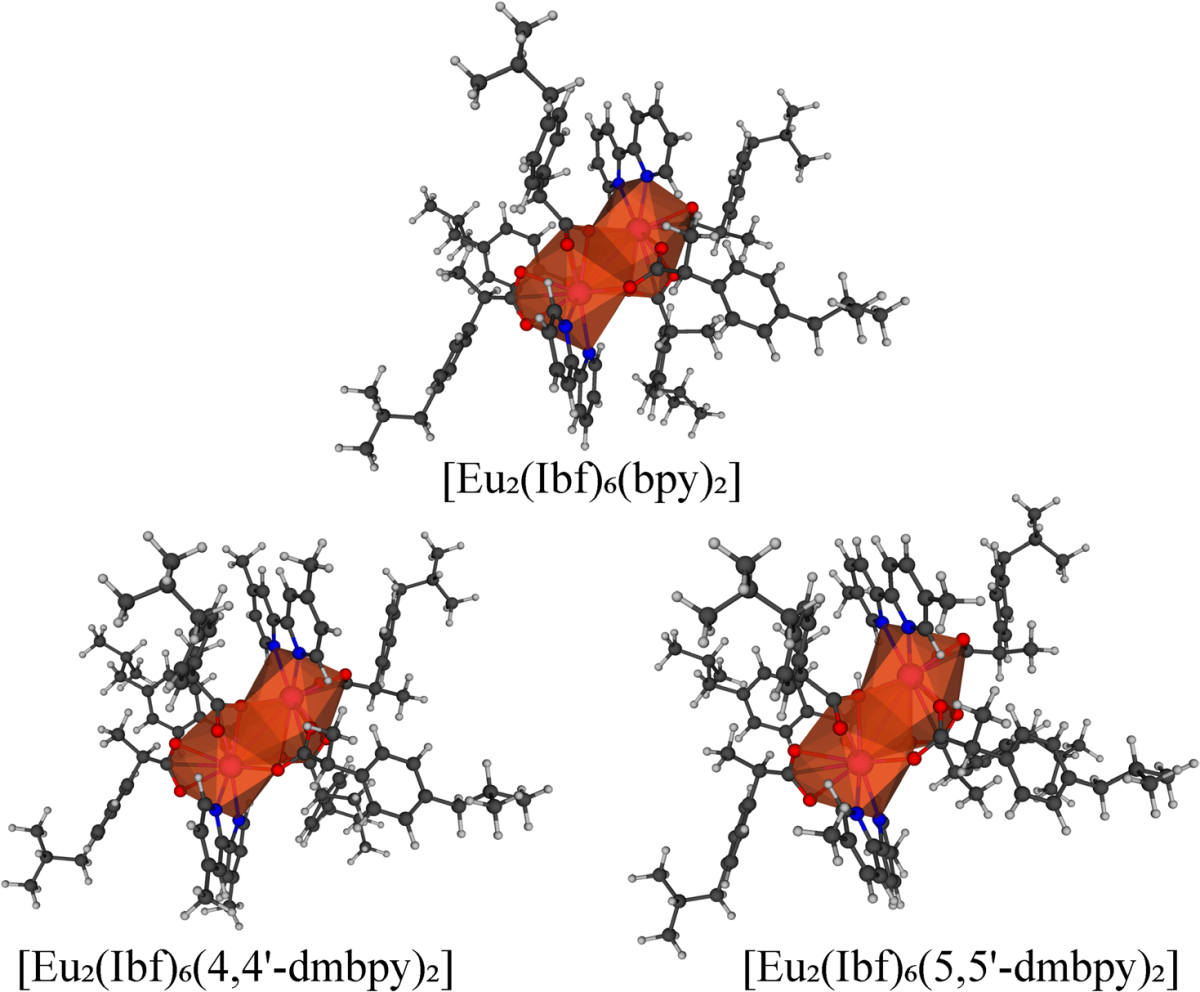
Structural representations of the three dinuclear Eu^3+^ complexes employed for parameterization and model validation: [Eu_2_(Ibf)_6_(bpy)_2_] (reference compound), [Eu_2_(Ibf)_6_(4,4′‐dmbpy)_2_], and [Eu_2_(Ibf)_6_(5,5′‐dmbpy)_2_]. The structural variation among these systems arises solely from the methyl substitution at the para (4,4′‐) or meta (5,5′‐) positions of the auxiliary bipyridine ring. bpy, 2,2′‐bipyridine; Ibf, ibuprofenate, 2‐(4‐isobutylphenyl)propanoate.

Following our prior study on the [Eu_2_(Ibf)_6_(bpy)_2_] complex [31], we later reported on two analogous systems: [Eu_2_(Ibf)_6_(4,4′‐dmbpy)_2_] and [Eu_2_(Ibf)_6_(5,5′‐dmbpy)_2_] [32]. As shown in Figure 1, the structural difference between them relies exclusively on the substitution of a hydrogen atom by a methyl group at the para and meta positions of the bipyridine ligand. Since the phenomenological intensity parameters were successfully obtained from the emission spectra of each system, we selected [Eu_2_(Ibf)_6_(bpy)_2_] for the model parameterization, using the [Eu_2_(Ibf)_6_(4,4′‐dmbpy)_2_] and [Eu_2_(Ibf)_6_(5,5′‐dmbpy)_2_] compounds to test its validity.

The parameterization was carried out using a custom‐built Python routine. We employed the generalized simulated annealing (GSA) nonlinear optimization algorithm to explore the multidimensional parameter space, seeking the optimal values for parameters defined in Table 1 that minimizes the following response function:

(12) $$F_{\text{resp}}=\left|\Omega_{2}^{\exp}-\Omega_{2}^{\text{calc}}\right|+\left|\Omega_{4}^{\exp}-\Omega_{4}^{\text{calc}}\right|$$

Following an extensive sampling of the multidimensional space, encompassing hundreds of thousands of objective function evaluations for the eight model parameters, the algorithm converged to a minimum value of 0.91. This optimized parameter set yielded relative errors of only 3.1% and 8.4% for the theoretical Ω_2_ and Ω_4_ intensity parameters, respectively, resulting in a mean percentage error of 5.8% (Table 2). The final parameter set is summarized in Table 3.

**TABLE 2:** Experimental and theoretical Judd–Ofelt intensity parameters (Ω_2_ and Ω_4_, ×10^−20^ cm^2^) for the reference complex [Eu_2_(Ibf)_6_(bpy)_2_], obtained from the semiempirical model parameterized via the generalized simulated annealing (GSA) algorithm.

Complex	Ω_2_ (Exp.)	Ω_2_ (Theo.)	Error (%)	Ω_4_ (Exp.)	Ω_4_ (Theo.)	Error (%)
[Eu_2_(Ibf)_6_(bpy)_2_]	7.73	7.97	3.1	7.98	8.65	8.4

**TABLE 3:** Optimized parameter set derived from the semiempirical model parameterization, employed for the purely theoretical calculation of Judd–Ofelt intensity parameters in Eu^3+^ complexes with Eu^3+^–O and Eu^3+^–N bonds.

Parameter	Value
^*ρ*^_0_ (O)	8.794063569891955
^*ρ*^_0_ (N)	36.8375804587622
*n* (O)	38.518952014370306
*n* (N)	1.3833921340661937
*ε* (O)	17.012836669370767
*ε* (N)	5.996909140771672
*D*	8.794063569891955
*C*	6.939504198187246

Using the parameter set presented in Table 3, we calculated the theoretical intensity parameters (Ω_2_ and Ω_4_) for the [Eu_2_(Ibf)_6_(4,4′‐dmbpy)_2_] and [Eu_2_(Ibf)_6_(5,5′‐dmbpy)_2_] complexes to validate our approach. A comparison with their corresponding experimental values (Table 4) reveals an outstanding agreement, with the proposed model yielding mean errors of only 1.7% and 3.9% for the Ω_2_ and Ω_4_, respectively. These findings highlight the robust predictive accuracy of our approach for lanthanide complexes sharing analogous coordination environments.

**TABLE 4:** Experimental and theoretical Judd–Ofelt intensity parameters (Ω₂ and Ω₄, ×10^−20^ cm^2^) for the analogous [Eu_2_(Ibf)_6_(4,4′‐dmbpy)_2_] and [Eu_2_(Ibf)_6_(5,5′‐dmbpy)_2_] complexes, obtained using the parameter set from Table 3.

Compound	Ω_2_ (Exp.)	Ω_2_ (Theo.)	Error (%)	Ω_4_ (Exp.)	Ω_4_ (Theo.)	Error (%)
[Eu_2_(Ibf)_6_(4,4′‐dmbpy)_2_]	8.39	8.12	3.2	8.57	8.49	0.93
[Eu_2_(Ibf)_6_(5,5′‐dmbpy)_2_]	9.14	9.13	0.11	8.73	8.14	6.8

*Note:* The resulting percentage errors demonstrate the transferability and predictive accuracy of the parameterized semiempirical model.

## 3 Theoretical Model for Nonradiative Emission Calculation

According to Bünzli [33], nonradiative decay encompasses various factors that interfere with the radiative decay process; therefore, there is no single phenomenology for this decay. However, vibrational decay is experimentally observed to play a highly significant role in nonradiative deactivation. Driven by this observation, a semiempirical model for vibronic decay was developed.

Furthermore, van Dijk and coworkers proposed an equation for the nonradiative decay rate based on Fermi's golden rule [24, 25]. To achieve this, the authors employed a nonadiabatic approximation for the electronuclear motion, which is represented by the perturbation Hamiltonian presented in Equation (13).

(13) $$\mathrm{\delta H}\left|\left.\Psi_{\mathrm{n\upsilon}}\right\rangle \right.=\sum_{A}\nabla_{A}\psi_{n}\cdot \nabla_{A}\chi_{\upsilon }$$

Utilizing the energy difference between the f–f levels (Δ*E*_0_) [25], the authors formulated the equation for nonradiative decay (*A*_NR_), which expresses a proportionality relationship between the two rates.

(14) $$A_{\mathrm{NR}}=\beta_{\mathrm{sym}}\frac{A_{R}}{{\Delta E}_{0}}e^{-\alpha \left({\Delta E}_{0}-2\hbar \omega_{M}\right)}$$

where

(15) $$A_{R}=\frac{4{\left|J\right|}^{2}}{3h^{4}c^{3}}{{\Delta E}_{0}}^{3}$$

The constant terms in Equation (15) can be grouped into a single term *C*_R_, since it depends only on the lanthanide ion, which in this case is Eu^3+^.

(16) $$C_{R}={\left(\frac{3h^{4}c^{3}}{4{\left|J\right|}^{2}}\right)}^{\frac{1}{3}}$$

Thus, Equation (15) can be rewritten in terms of the ${\Delta E}_{0}$ parameter.

(17) $${\Delta E}_{0}=C_{R}\sqrt[{3}]{A_{R}}$$

Substituting the ${\Delta E}_{0}$ term into Equation (14) yields:

(18) $$A_{\mathrm{NR}}=\beta_{\mathrm{sym}}\frac{A_{R}}{C_{R}\;\sqrt[{3}]{A_{R}}}e^{-\alpha \left(C_{R}\;\sqrt[{3}]{A_{R}}-2\hbar \omega_{M}\right)}$$

(19) $$A_{\mathrm{NR}}=\frac{\beta_{\mathrm{sym}}}{C_{R}}{A_{R}}^{\frac{2}{3}}\;e^{-\alpha \left(C_{R}\;\sqrt[{3}]{A_{R}}-2\hbar \omega_{M}\right)}$$

Applying the Taylor series expansion approximation, one obtains:

(20) $$A_{\mathrm{NR}}=\frac{\beta_{\mathrm{sym}}}{C_{R}}{A_{R}}^{\frac{2}{3}}\;\left[1-\alpha \left(C_{R}\sqrt[{3}]{A_{R}}-2\hbar \omega_{M}\right)\right]$$

Rearranging Equation (20) yields:

(21) $$A_{\mathrm{NR}}={A_{R}}^{\frac{2}{3}}\left[\frac{\beta_{\mathrm{sym}}}{C_{R}}-\alpha \frac{\beta_{\mathrm{sym}}}{C_{R}}\left(C_{R}\sqrt[{3}]{A_{R}}-2\hbar \omega_{M}\right)\right]$$

Doing $\beta_{s}=\frac{\beta_{\mathrm{sym}}}{C_{R}}$ and $\alpha_{s}=\alpha \frac{\beta_{\mathrm{sym}}}{C_{R}}$

(22) $$A_{\mathrm{NR}}={A_{R}}^{\frac{2}{3}}\left[\beta_{s}-\alpha_{s}\left(C_{R}\sqrt[{3}]{A_{R}}-2\hbar \omega_{M}\right)\right]$$

To introduce additional degrees of freedom into the model and account for the discrepancy between the phenomenological radiative emission rate (*A*_R_) derived from Equation (14) and the theoretically predicted rate according to Equation (24), we proposed the adjustment detailed in Equation (24).

(23) $$A_{R}=\frac{64\pi^{4}\nu^{3}}{3h\left(2J+1\right)}\left[\frac{n\left(n^{2}+2\right)}{9}S_{\mathrm{ed}}+n^{3}S_{\mathrm{md}}\right]$$

where *h* is the Planck constant, 2*J* + 1 is the degeneracy of the initial state, and *n* is the refractive index of the medium, usually assumed to be equal to 1.5. *S*_ed_ and *S*_md_ in Equation (23) are the forced electric dipole and magnetic dipole line strengths, respectively.

(24) $${A_{R}}^{\mathrm{mod}}=\left|aA_{R}-b\right|$$

where *a* and *b*, together with the $\alpha_{s}$ and $\beta_{s}$, were treated as parameters of the proposed model. By substituting $A_{R}$ with ${A_{R}}^{\mathrm{mod}}$ in Equation (22), we obtain Equation (25).

(25) $$A_{\mathrm{NR}}={\left({A_{R}}^{\mathrm{mod}}\right)}^{\frac{2}{3}}\;\left[\beta_{s}-\alpha_{s}\left(C_{R}{\left({A_{R}}^{\mathrm{mod}}\right)}^{\frac{1}{3}}-2\hbar \omega_{M}\right)\right]$$

The parameterization process is described below.

### 3.1 Parameterization

The parameterization procedure for the model (Equation 25) mirrored the procedure employed for the intensity parameters. However, the objective function ($F_{\text{resp}}$) minimized during this step was defined as follows:

(26) $$F_{\text{resp}}=\left|A_{\mathrm{NR}}^{\exp}-A_{\mathrm{NR}}\right|$$

Because the proposed model (Equation 25) describes a continuous parametric function, its parameterization required a simultaneous fit across all three investigated systems: [Eu_2_(Ibf)_6_(bpy)_2_], [Eu_2_(Ibf)_6_(4,4′‐dmbpy)_2_], and [Eu_2_(Ibf)_6_(5,5′‐dmbpy)_2_]. Following an extensive sampling of the parameter space, the optimization algorithm converged to an exceptional fit, yielding a mean error of only 0.068% for the nonradiative decay rate (*A*_NR_).

However, to ensure stable convergence of parametrization, it was necessary to use all three available complexes simultaneously as the training set. A LOO procedure with *n* = 3 would reduce the dataset to only two points, which is insufficient to uniquely determine the four model parameters, resulting in an underdetermined system.

The optimized parameter set and the corresponding theoretical results for the three complexes are summarized in Tables 5 and 6, respectively.

**TABLE 5:** Optimized parameter set of the semiempirical vibronic model for the purely theoretical calculation of the nonradiative emission rate (*A*_NR_) in Eu^3+^ complexes.

Parameter	Value
*a*	10
*b*	4.3568 × 10^3^
$\alpha_{s}$	−1.85371 × 10^−3^
$\beta_{s}$	1.4347 × 10^3^

**TABLE 6:** Experimental and theoretical nonradiative emission rates (*A*_NR_) for the [Eu_2_(Ibf)_6_(bpy)_2_], [Eu_2_(Ibf)_6_(4,4′‐dmbpy)_2_], and [Eu_2_(Ibf)_6_(5,5′‐dmbpy)_2_] complexes, obtained with the parameterized semiempirical vibronic model. The percentage errors confirm the accuracy of the model.

Compound	$A_{\mathrm{NR}}^{\exp}$ (s^−1^)	$A_{\mathrm{NR}}$ (s^−1^)	Error (%)
[Eu_2_(Ibf)_6_(bpy)_2_]	176.92	176.92	0.000
[Eu_2_(Ibf)_6_(4,4′‐dmbpy)_2_]	59.29	59.30	0.015
[Eu_2_(Ibf)_6_(5,5′‐dmbpy)_2_]	85.08	84.92	0.188

Beyond facilitating the theoretical determination of *A*_NR_, the development of this model enables the direct calculation of both the theoretical lifetime (*τ*) and the quantum efficiency (η), as defined by Equations (27) and (28), respectively.

(27) $$\tau =\frac{1}{A_{R}+A_{\mathrm{NR}}}$$

(28) $$\eta =\frac{A_{R}}{A_{R}+A_{\mathrm{NR}}}$$

Table 7 presents the theoretical and experimental photophysical parameters for the [Eu_2_(Ibf)_6_(bpy)_2_], [Eu_2_(Ibf)_6_(4,4′‐dmbpy)_2_], and [Eu_2_(Ibf)_6_(5,5′‐dmbpy)_2_] systems. An initial assessment reveals a remarkable agreement between the experimental data and the theoretical predictions across all three complexes. Regarding the radiative rate (*A*_R_), the most deviation (approximately 4.8%) was observed for the [Eu_2_(Ibf)_6_(bpy)_2_] complex, whereas the errors for the other systems remained strictly below 1.5%. Furthermore, the proposed model for *A*_NR_ demonstrated an exceptional parameterization quality, yielding a maximum error of merely 0.19% for the [Eu₂(Ibf)_6_(5,5′‐dmbpy)_2_] analog. This high accuracy in predicting *A*_NR_ consequently allowed for a highly reliable estimation of the emission lifetime (τ). For this parameter, the largest deviation was 3.4% for the [Eu_2_(Ibf)_6_(bpy)_2_] complex, while a smallest error (0.48%) was achieved for [Eu_2_(Ibf)_6_(4,4′‐dmbpy)_2_]. Finally, the intrinsic quantum efficiency (η) was predicted with remarkable precision, yielding an average error of merely 0.4% across the three investigated complexes.

**TABLE 7:** Comparison of the experimental and calculated photophysical parameters, including radiative (*A*_*R*_) and nonradiative (*A*_*NR*_) emission rates, ^5^D_0_ excited‐state lifetime (τ), and intrinsic quantum efficiency (η) for the [Eu_2_(Ibf)_6_(bpy)_2_], [Eu_2_(Ibf)_6_(4,4′‐dmbpy)_2_], and [Eu_2_(Ibf)_6_(5,5′‐dmbpy)_2_] complexes.

Compound	*A*_R_ (s^−1^)		*A*_NR_ (s^−1^)		τ (ms)		η (%)	
	Exp.	Theo.	Exp.	Theo.	Exp.	Theo.	Exp.	Theo.
[Eu_2_(Ibf)_6_(bpy)_2_]	401.12	420.25	176.92	176.92	1.73	1.67	69.39	70.37
[Eu_2_(Ibf)_6_(4,4′‐dmbpy)_2_]	423.80	421.48	59.29	59.30	2.07	2.08	87.73	87.66
[Eu_2_(Ibf)_6_(5,5′‐dmbpy)_2_]	452.55	446.58	85.08	84.92	1.86	1.88	84.17	84.02

## 4 Design of New Luminescent Compounds Based on [Eu_2_(Ibf)_6_(bpy)_2_] Complex

As previously discussed, the design protocol adheres to a four‐step protocol, with the final stage dedicated to evaluating the luminescent properties of the engineered systems. This stage comprises four sequential computational steps: (i) determination of the Judd–Ofelt intensity parameters; (ii) calculation of the energy transfer and back‐transfer rates; (iii) computation of the radiative and nonradiative emission rates; and (iv) evaluation of the intrinsic quantum efficiency and overall quantum yield.

The absence of analytical equations for calculating the charge factors and polarizabilities of metal–ligand bonds prevents the direct theoretical determination of the intensity parameters (step i). To overcome this limitation, we rely on the assumption that the newly designed systems will exhibit coordination polyhedra structurally analogous to the [Eu_2_(Ibf)_6_(bpy)_2_] reference complex. Under this premise, the semiempirical models developed herein can be successfully employed to evaluate these photophysical properties. In light of this constraint, we designed eight new systems for this study, ensuring their coordination polyhedra closely resemble those of the complexes used to parameterize the model. These eight structures were generated through the systematic substitution of the methyl groups at the meta and para positions of the bipyridine ligand with the following substituents: NH_2_, NO_2_, OCH_3_, and OH.

**FIGURE 2:**
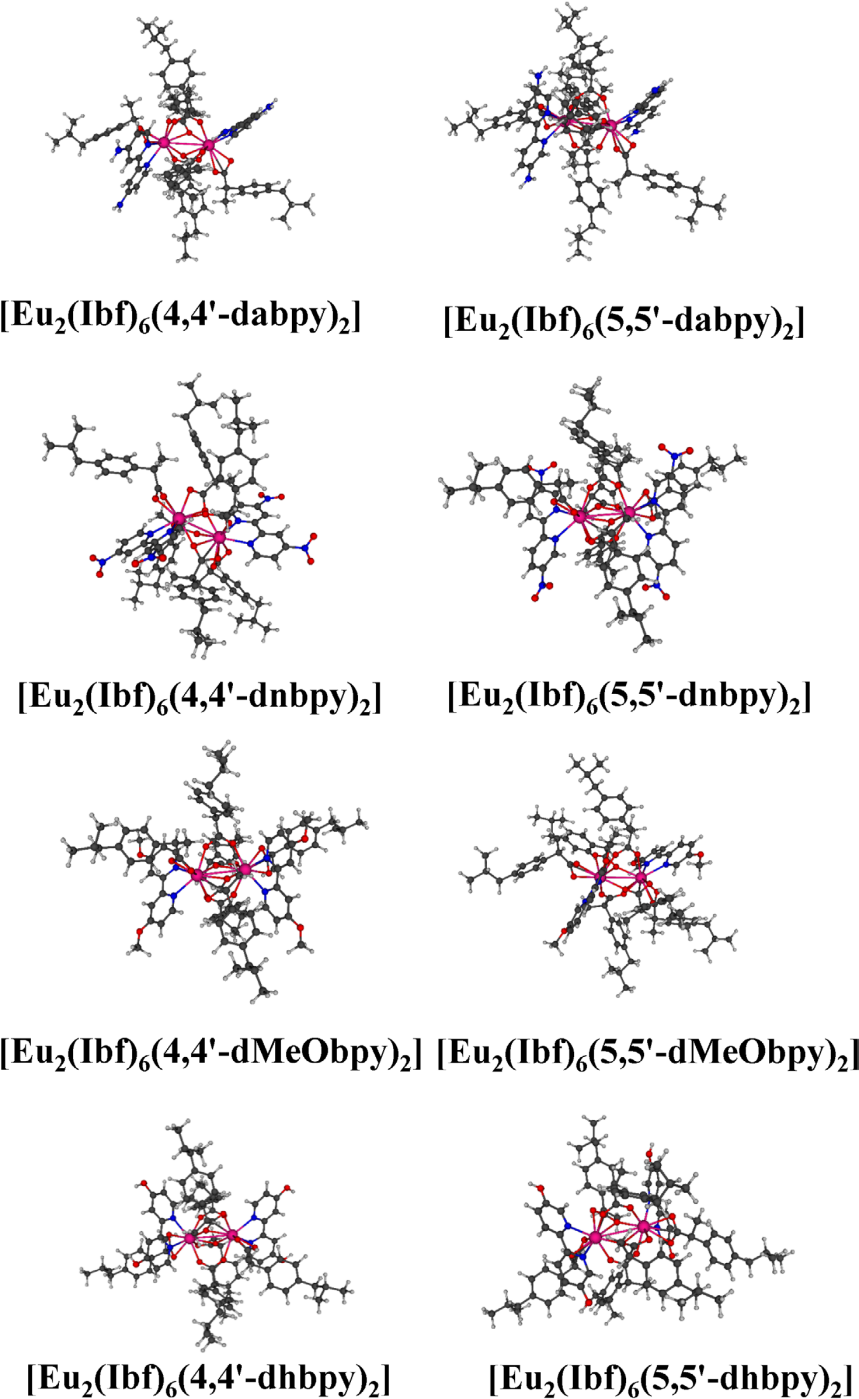
Ground‐state geometries of the eight newly proposed dinuclear Eu^3+^ complexes, optimized at the RM1 semiempirical level. The structures were derived from [Eu_2_(Ibf)_6_(4,4′‐dmbpy)_2_] and [Eu_2_(Ibf)_6_(5,5′‐dmbpy)_2_] by replacing the methyl groups with NH_2_ (dabpy), NO_2_ (dnbpy), OCH_3_ (dMeObpy), or OH (dhbpy) at the 4,4′‐and 5,5′‐positions of the 2,2′‐bipyridine ligand.

The three‐dimensional geometries of the eight novel complexes were generated by systematically modifying the previously optimized reference structures [Eu_2_(Ibf)_6_(4,4′‐dmbpy)_2_] and [Eu_2_(Ibf)_6_(5,5′‐dmbpy)_2_] [32]. The respective substituent groups were introduced using the Avogadro software (version 1.2.0) [34, 35]. Subsequently, full geometry optimizations were performed using the RM1 semiempirical model [8] implemented in the MOPAC program (version 18.117W) [36]. The calculations employed the following keywords: RM1 PRECISE GEO‐OK XYZ T = 10D ALLVEC BFGS GNORM = 0.25 CHARGE = 0. The resulting optimized structures are depicted in Figure 2.

Following the optimized structures, the comprehensive photophysical profiles of the eight novel complexes were evaluated. The models developed herein for the intensity parameters and nonradiative emission rates were implemented via custom Python routines and applied to these new systems. All subsequent photophysical properties were computed using the LUMPAC program (version 2.0) [37].

The Judd–Ofelt intensity parameters (Ω_λ_) are the fundamental descriptors of the interaction between the ligand field and the 4f states of Eu^3+^. Within the spectroscopic formalism of lanthanides, Ω_2_ is recognized as the parameter most sensitive to the symmetry of the coordination environment and the covalency of the metal–ligand bonds [38, 39], whereas Ω_4_ and Ω_6_ are more closely associated with the rigidity and dielectric nature of the surroundings [27]. An analysis of Table 8 reveals that the Ω_2_ parameter ranges from 7.86 × 10^−20^ cm^2^ for the 4,4′‐dhbpy analog to 10.18 × 10^−20^ cm^2^ for the 5,5′‐dnbpy system. The higher Ω_2_ value for the [Eu_2_(Ibf)_6_(5,5′‐dnbpy)_2_] complex is physically consistent due to the strongly electron‐withdrawing nitro group contributing to an increased polarization of the Eu‐O (from Ibf) bonds via an inductive effect. This, in turn, enhances the odd‐parity crystal field component, which primarily governs the magnitude of Ω_2_. Furthermore, the asymmetry of the ligand field induced by the –NO_2_ group at the meta position is likely greater than that observed for the para substitution, thereby promoting stronger configuration mixing in the 4f states. Overall, the Ω_2_ values across this series (7.86–10.18 × 10^−20^ cm^2^) exceed that of the reference complex, confirming that both the nature and the position of the substituents effectively modulate the ligand field around the Eu^3+^ ion. As expected, the Ω_4_ parameter exhibits less significant variation (7.96–8.94 × 10^−20^ cm^2^), reflecting its lower sensitivity to modifications in the second coordination sphere. Finally, Ω_6_ remains consistently very low (~0.06–0.07 × 10^−20^ cm^2^) for all systems, a typical behavior for Eu^3+^ complexes where the ^5^D_0_ → ^7^F_6_ transition has a negligible contribution to the overall emission spectrum.

**TABLE 8:** Theoretical calculated Judd–Ofelt intensity parameters (Ω_2_, Ω_4_, and Ω_6_), radiative (*A*_R_) and nonradiative (*A*_NR_) emission rates, ^5^D_0_ excited‐state lifetime (τ), and intrinsic quantum efficiency (η) for the eight newly proposed Eu^3+^ systems.

Complex	Ω_λ_ (×10^−20^ cm^2^)			*A*_R_ (s^−1^)	*A*_NR_ (s^−1^)	τ (ms)	η (%)
	Ω_2_	Ω_4_	Ω_6_				
[Eu_2_(Ibf)_6_(4,4′‐dabpy)_2_]	7.95	8.15	0.06	411.51	123.11	1.8705	76.97
[Eu_2_(Ibf)_6_(5,5′‐dabpy)_2_]	9.13	8.20	0.06	447.67	57.15	1.9809	88.68
[Eu_2_(Ibf)_6_(4,4′‐dnbpy)_2_]	8.06	8.94	0.06	426.80	44.41	2.1222	90.57
[Eu_2_(Ibf)_6_(5,5′‐dnbpy)_2_]	10.18	8.85	0.06	489.04	276.89	1.3056	63.85
[Eu_2_(Ibf)_6_(4,4′‐dMeObpy)_2_]	8.47	8.48	0.06	431.96	17.76	2.2236	96.05
[Eu_2_(Ibf)_6_(5,5′‐dMeObpy)_2_]	8.70	7.96	0.07	431.13	22.67	2.2036	95.00
[Eu_2_(Ibf)_6_(4,4′‐dhbpy)_2_]	7.86	8.58	0.06	415.33	104.36	1.9242	79.92
[Eu_2_(Ibf)_6_(5,5′‐dhbpy)_2_]	9.60	8.45	0.06	465.56	150.39	1.6235	75.58

The radiative emission rate exhibits a strong positive correlation with Ω_2_. Specifically, the complex with the highest Ω_2_, [Eu_2_(Ibf)_6_(5,5′‐dnbpy)_2_], also presents the highest *A*_R_ (489.04 s^−1^), while the system with the lowest Ω_2_, [Eu_2_(Ibf)_6_(4,4′‐dhbpy)_2_], displays the lowest *A*_R_ (415.33 s^−1^). This trend is theoretically expected, given that the ^5^D_0_ → ^7^F_2_ transition is a hypersensitive forced electric dipole transition, governed by Ω_2_, and provides the primary contribution to *A*_R_ in Eu^3+^ complexes. Consequently, substituents that maximize Ω_2_ directly enhance the overall radiative emission rate of the system.

Among the photophysical parameters detailed in Table 8, the nonradiative emission rate exhibits the most pronounced variation, ranging from 17.76 s^−1^ for [Eu_2_(Ibf)_6_(4,4′‐dMeObpy)_2_] to 276.89 s^−1^ for [Eu_2_(Ibf)_6_(5,5′‐dnbpy)_2_]. This broad range highlights the acute sensitivity of the multiphonon relaxation mechanism to both the electronic nature and position of the substituents. Notably, complexes featuring electron‐donating groups in the para position display the lowest nonradiative rates within the series.

This behavior can be rationalized by the distance‐dependent nature of the multiphonon coupling. Although the –OCH_3_ and –OH groups possess high frequency C–H and O–H oscillators, respectively, their placement at the para position maximizes their distance from the metal center, thereby significantly attenuating nonradiative losses. Notably, the –OCH_3_ group at the para position is more effective than –OH group in suppressing nonradiative decay. This suggests that replacing the acidic hydrogen with a methoxy group plays a critical role in eliminating the primary deactivation channel associated with the O–H oscillator (~3600 cm^−1^), which is notorious for efficiently quenching the ^5^D_0_ emitting level. Conversely, the [Eu_2_(Ibf)_6_(5,5′‐dnbpy)_2_] complex exhibits the highest nonradiative rate (276.89 s^−1^). The introduction of the –NO_2_ group at the meta position not only places the N=O oscillators in closer spatial proximity to the Eu^3+^ center, but also introduces vibrational modes (N–O: ~1300–1550 cm^−1^) that act as highly efficient acceptors in the multiphonon relaxation process.

The lifetime (τ) is governed by the interplay between the radiative and nonradiative decay rates. The predicted values range from 1.31 ms for the [Eu_2_(Ibf)_6_(5,5′‐dnbpy)_2_] complex to 2.22 ms for the [Eu_2_(Ibf)_6_(4,4′‐dMeObpy)_2_] analog. Notably, all calculated lifetimes are longer than those typically observed for Eu^3+^ complexes containing water coordinated molecules [40, 41]. This confirms the effective shielding of the ion by a coordination environment predominantly composed of carboxylate and bipyridine ligands.

The highest intrinsic quantum efficiency (η) values are achieved by dMeObpy‐functionalized complexes (η > 94%), irrespective of the substitution position. This indicates that, among the evaluated substituents, the methoxy group is the most effective at maximizing η, primarily by suppressing nonradiative decay pathways. Conversely, the [Eu_2_(Ibf)_6_(5,5′‐dnbpy)_2_] complex presents a distinctly contrasting photophysical profile: it simultaneously exhibits the highest *A*_R_ (489.04 s^−1^) and the highest *A*_NR_ (276.89 s^−1^) in the series. This competition results in a moderate η of 63.85% and the shortest emission lifetime (τ = 1.31 ms). Although this system possesses the most radiatively active metal center, its intrinsic efficiency is substantially bottlenecked by pronounced nonradiative losses. Nevertheless, this scenario, where an intense emission potential (high *A*_R_) is partially offset by efficient nonradiative deactivation, can be highly advantageous for specific high‐luminance device applications, where a rapid absolute photon emission rate is prioritized over a prolonged excited‐state lifetime.

The energies of the singlet and triplet excited states were predicted at the configuration interaction simple (CIS) level, employing the INDO/S (intermediate neglect of differential overlap/spectroscopic) approach [42, 43], as implemented in the ORCA program (version 5.0.2) [44]. To model the trivalent lanthanide, the Eu^3+^ center was represented by a +3e point charge. The CIS calculations were performed within an excitation window comprising the 20 highest occupied and 20 lowest unoccupied molecular orbitals, which corresponds to the standard default setting of the LUMPAC software. The resulting singlet and triplet excited state energies, alongside the *R*_L_ values, are summarized in Table 9.

**TABLE 9:** Calculated singlet (S_1_) and triplet (T_1_) excited‐state energies (cm^−1^) and energy donor and acceptor center (*R*_L_, Å) for the eight newly proposed Eu^3+^ complexes, obtained at the INDO/S‐CIS level of theory.

Complex	Triplet (cm^−1^)	*R*_L_ (Å)	Singlet (cm^−1^)	*R*_L_ (Å)
[Eu_2_(Ibf)_6_(4,4′‐dabpy)_2_]	22,277.4	8.2494	34,564.6	4.1147
[Eu_2_(Ibf)_6_(5,5′‐dabpy)_2_]	21,145.7	3.8700	30,250.1	5.4699
[Eu_2_(Ibf)_6_(4,4′‐dnbpy)_2_]	22,745.1	5.6361	25,175.9	6.4484
[Eu_2_(Ibf)_6_(5,5′‐dnbpy)_2_]	22,499.0	6.5405	24,164.8	8.2866
[Eu_2_(Ibf)_6_(4,4′‐dMeObpy)_2_]	22,255.4	4.0767	33,484.6	4.0730
[Eu_2_(Ibf)_6_(5,5′‐dMeObpy)_2_]	22,856.6	4.0448	32,489.9	5.4743
[Eu_2_(Ibf)_6_(4,4′‐dhbpy)_2_]	22,595.6	6.0756	34,292.1	5.2564
[Eu_2_(Ibf)_6_(5,5′‐dhbpy)_2_]	22,288.3	4.0495	32,405.8	4.9682

The calculated triplet state (T_1_) energies for the eight systems range from 21,146 cm^−1^ (5,5′‐dabpy) to 22,857 cm^−1^ (5,5′‐dMeObpy). All T_1_ levels lie above the Eu^3+ 5^D_0_ emitting state (~17,400 cm^−1^), presenting an energy gap greater than 3700 cm^−1^. This large separation is highly favorable for preventing back‐transfer from the ^5^D_0_ level. However, a closer inspection reveals that the [Eu_2_(Ibf)_6_(5,5′‐dabpy)_2_] complex exhibits the lowest T_1_ in the series (21,146 cm^−1^), which positions it in closer proximity to the ^5^D_1_ level (~19,000 cm^−1^). This reduced energy gap likely intensifies the competition between the forward energy transfer to ^5^D_1_ and the subsequent back‐transfer from ^5^D_1_ via thermally activated reverse excitation. This photophysical bottleneck is perfectly consistent with the relatively lower sensitization efficiency (87.20%, Table 11) observed for this complex when compared to its 5,5′‐substituted counterparts.

In contrast, the incorporation of the ‐NO_2_ substituents results in a substantial stabilization of the singlet states (S_1_), yielding values of 25,176 cm^−1^ (4,4′‐dnbpy) and 24,165 cm^−1^ (5,5′‐dnbpy). This trend aligns seamlessly with the nature of electron‐withdrawing groups, which stabilize the LUMO and consequently reduce the HOMO–LUMO gap. As a result, the singlet–triplet energy gap in these nitro‐functionalized complexes is notably compressed to 1665–2430 cm^−1^, compared to the broader 9100–12,300 cm^−1^ range seen with other substituents. Although this reduced gap may facilitate intersystem crossing (ISC), it simultaneously elevates the risk of competitive excited‐state deactivation through internal conversion.

Using the singlet and triplet state energies, together with the corresponding distance *R*_L_ reported in Table 9, the ligand–metal energy rates were estimated with the LUMPAC 2.0 software [37]. These rates were computed using the theoretical framework proposed by Malta and co‐workers [43, 44, 45] which consider two mechanisms: the direct Coulombic interaction (CI) mechanism and the exchange (Ex.) mechanism, described by Equations (29) and (31), respectively.

(29) $$\begin{array}{c} W_{\mathrm{ET}}^{\mathrm{CI}}=\frac{2\pi }{\hbar }\frac{e^{2}S_{L}F}{G\left(2J+1\right)}\sum_{\lambda =2,4,6}\Lambda_{\lambda }{\left\langle \psi^{\prime }J^{\prime }\left\|U^{\left(\lambda \right)}\right\|\mathrm{\psi J}\right\rangle }^{2} \end{array}$$

(30) $$\begin{array}{c} \begin{array}{c} \Lambda_{\lambda }=2\Omega_{\lambda }^{\mathrm{FED}}{\left(1-\sigma_{1}\right)}^{2}\left(\frac{1}{R_{L}^{6}}\right)+{\left\langle r^{\lambda }\right\rangle }^{2}{\left\langle 3\left\|C^{\left(\lambda \right)}\right\|3\right\rangle }^{2}{\left(1-\sigma_{\lambda }\right)}^{2}\left(\frac{\lambda +1}{{\left(R_{L}^{\lambda +2}\right)}^{2}}\right) \end{array} \end{array}$$

(31) $$\begin{array}{c} W_{\mathrm{ET}}^{\mathrm{CI}}=\frac{8\pi }{3\hbar }\frac{e^{2}}{R_{L}^{4}}\frac{{\left(1-\sigma_{0}\right)}^{2}F}{G\left(2J+1\right)}{\left\langle \psi^{\prime }J^{\prime }\left\|S\right\|\mathrm{\psi J}\right\rangle }^{2}\\ \;\sum_{m}{\left\langle \Psi_{N-1}\Pi \left\|\sum_{j}C_{0}^{\left(1\right)}\left(j\right)\;s_{-m}\left(j\right)\right\|\Psi_{N-1}\Pi^{*}\right\rangle }^{2} \end{array}$$

The dependence of these expressions on the ligand state energies is contained in the *F* term, defined as follows:

(32) $$F=\frac{1}{\hbar \gamma_{L}}\sqrt{\frac{\ln2}{\pi }}e^{-{\left(\frac{\Delta }{\hbar \gamma_{L}}\right)}^{2}\ln2}$$

where *Δ* represents the energy gap between the ligand donor state and the accepting excited state of the lanthanide ion. This formulation assumes that the $\gamma_{L}$ ligand bandwidth is significantly larger than the width of the 4f–4f transitions of the Ln(III) ion.

The donor–acceptor distance *R*_L_ is determined according to:

(33) $$R_{L}=\frac{\sum_{i}c_{i}^{2}R_{L_{i}}}{\sum_{i}c_{i}^{2}}$$

where *R*_min_ corresponds to the shortest lanthanide and a directly coordinated atom. The *R*_L_ value obtained using the molecular orbital coefficient of atom *i* (*c*_*i*_) in the electronic state of interest, together with the corresponding distance *R*_*L,i*_ between atom *i* and the Ln(III) ion. The coefficients ci were obtained from the INDO/S‐CIS calculations, while the *R*_*L,i*_ distances were extracted from the geometry employed in these calculations.

The *R*_L_ values, correspond to the distance between the acceptor and donor states in the energy transfer process, are relevant for evaluating the energy transfer rates via the Dexter and Förster mechanisms. This parameter is also used to estimate the $\left(1-\sigma_{0}\right)$ shielding factor, following the revised model proposed by Malta's group in 2019 [22].

(34) $$\left(1-\sigma_{0}\right)\approx 0.05{\left(\frac{R_{\min}}{R_{L}}\right)}^{\frac{7}{2}}$$

The ${\left\langle \psi^{\prime }J^{\prime }\left\|U^{\left(\lambda \right)}\right\|\mathrm{\psi J}\right\rangle }^{2}$ and ${\left\langle \psi^{\prime }J^{\prime }\left\|S\right\|\mathrm{\psi J}\right\rangle }^{2}$ reduced matrix elements appearing in Equations (29) and (31) are responsible for defining the selection rules associated with the total angular momentum *J*. For the direct Coulombic mechanism, transitions with |*ΔJ*| = 2, 4, 6 are allowed, whereas for the exchange mechanisms, transition with |*ΔJ*| = 0, 1 (excluding *J* = *J*′ = 0) are permitted. The values of ${\left\langle \psi^{\prime }J^{\prime }\left\|U^{\left(\lambda \right)}\right\|\mathrm{\psi J}\right\rangle }^{2}$ tabulated by Carnall et al. [46] and those estimated by Kasprzycka and coworkers [47] for ${\left\langle \psi^{\prime }J^{\prime }\left\|U^{\left(\lambda \right)}\right\|\mathrm{\psi J}\right\rangle }^{2}$, both implemented in LUMPAC, were employed in the calculations. The energies of the ^7^F_0_, ^7^F_1_, ^5^D_0_, ^5^D_1_, and ^5^D_4_ states of Eu(III) were taken from Carnall et al. [46] The back‐transfer (*W*_BET_) rate was estimated by multiplying the corresponding forward energy transfer rate by the Boltzmann factor, $e^{-\frac{\left|∆\right|}{k_{B}T}}$, where *T* is the temperature (300 K) and *k*_B_ is the Boltzmann constant.

The forced electric‐dipole (FED) intensity parameters ($\Omega_{\lambda }^{\mathrm{FED}}$) required for the CI mechanism were evaluated considering only the contribution of Equation (3) for the calculation of $\Omega_{\lambda }$. All other quantities involved in Equations ((29), (30), (31)–(29), (30), (31)) are described in detail in the literature [22, 45, 48].

**FIGURE 3:**
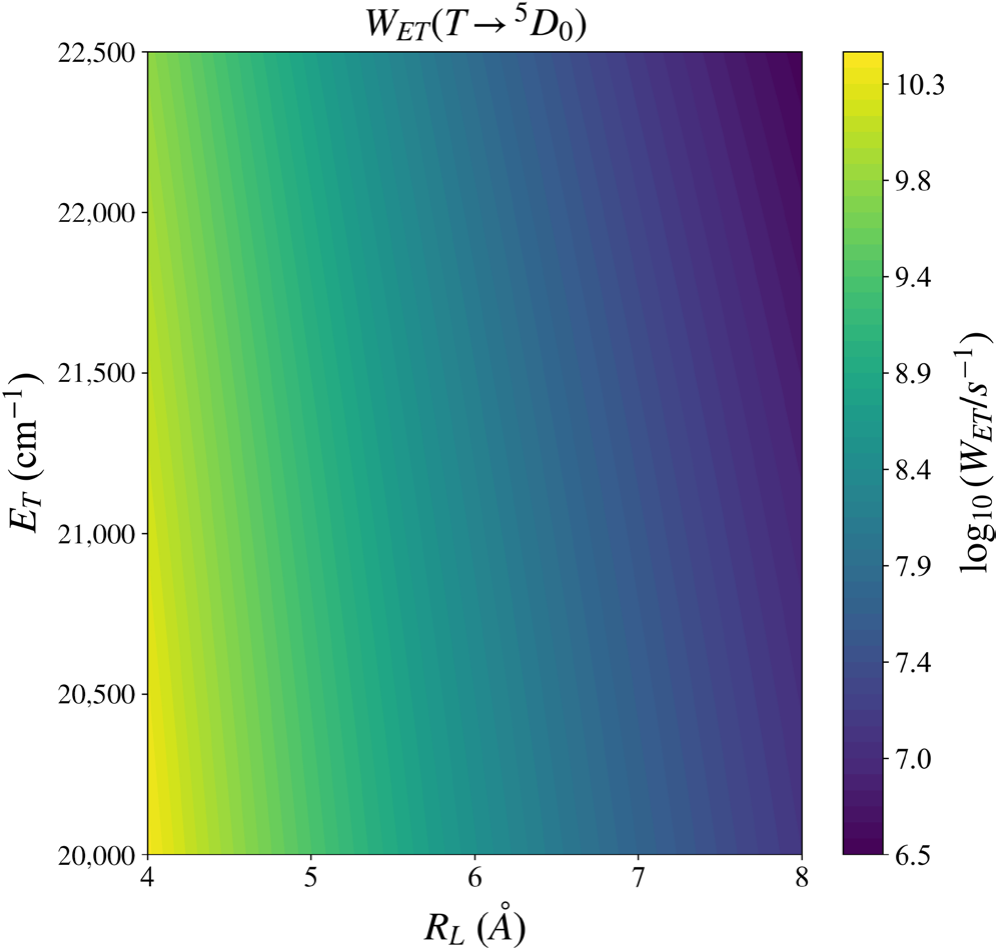
Dependence of the ligand‐Eu^3+^ energy transfer rate [*W*_ET_(T → ^5^D_0_)] on the donor–acceptor distance *R*_L_ and triplet‐state energy (*E*_T_). The color scale represents log_10_(*W*_ET_/s^−1^).

A systematic analysis of the energy transfer rate (*W*_ET_) for the T → ^5^D_0_ channel, which is the most relevant pathway for Eu^3+^ excitation, as a function of *R*_L_ (4.0–8.0 Å) and of the triplet‐state energy (20,000–22,500 cm^−1^) (Figure 3), shows that the magnitude of *W*_ET_ is primarily governed by *R*_L_. The rate spans more than six orders of magnitude as *R*_L_ increases. For *R*_L_≈4.0 Å, the predominance of the exchange mechanism leads to high rates in the range of 10^9^–10^10^ s^−1^, whereas increasing *R*_L_ to 8.0 Å lowers the magnitude to 10^5^–10^6^ s^−1^. The triplet‐state energy acts as a secondary modulator that tunes the efficiency within each order of magnitude. Energies in the 20,000–21,500 cm^−1^ range maximize the Franck–Condon factor (Equation 32), while energies above 22,000 cm^−1^ induce a gradual decrease of the rate due to the enlargement of the energy gap.

Table 10 presents the computed energy transfer (*W*_ET_) and back‐transfer (*W*_BT_) rates for the three main sensitization pathways. For the entire series of eight complexes, the T_1_ → ^5^D_0_ transition acts as the primary energy transfer pathway, secondarily supported by the T_1_ → ^5^D_1_ channel, while S_1_ → ^5^D_4_ remains largely negligible. This functional hierarchy is consistent with the well‐established antenna effect in Eu^3+^ complexes, wherein the S_1_ → T_1_ intersystem crossing acts as the main sensitization event. Given the theoretical triplet energies evaluated herein (21,146–22,857 cm^−1^, Table 9), the T_1_ → ^5^D_0_ pathway naturally fulfills the optimal resonance condition for efficient energy transfer.

**TABLE 10:** Calculated ligand–metal energy transfer (*W*_ET_) and back‐transfer (*W*_BT_) rates governing the overall sensitization process in the eight rationally designed Eu^3+^ complexes.

Complex	*W*_ET_ (s^−1^)		*W*_BT_ (s^−1^)	
[Eu_2_(Ibf)_6_(4,4′‐dabpy)_2_]	S_1_ → ^5^D_4_	9.8171 × 10^0^	^5^D_4_ → S_1_	1.3793 × 10^−15^
	T_1_ → ^5^D_1_	1.7430 × 10^6^	^5^D_1_ → T_1_	1.8490 × 10^−1^
	T_1_ → ^5^D_0_	3.4216 × 10^6^	^5^D_0_ → T_1_	1.4913 × 10^−5^
[Eu_2_(Ibf)_6_(5,5′‐dabpy)_2_]	S_1_ → ^5^D_4_	6.6481 × 10^3^	^5^D_4_ → S_1_	1.3422 × 10^−3^
	T_1_ → ^5^D_1_	2.1077 × 10^5^	^5^D_1_ → T_1_	1.5137 × 10^0^
	T_1_ → ^5^D_0_	5.2751 × 10^5^	^5^D_0_ → T_1_	1.5566 × 10^−4^
[Eu_2_(Ibf)_6_(4,4′‐dnbpy)_2_]	S_1_ → ^5^D_4_	1.7553 × 10^3^	^5^D_4_ → S_1_	6.0199 × 10^5^
	T_1_ → ^5^D_1_	9.7106 × 10^3^	^5^D_1_ → T_1_	9.2798 × 10^−6^
	T_1_ → ^5^D_0_	1.4531 × 10^4^	^5^D_0_ → T_1_	5.7054 × 10^−10^
[Eu_2_(Ibf)_6_(5,5′‐dnbpy)_2_]	S_1_ → ^5^D_4_	6.0180 × 10^3^	^5^D_4_ → S_1_	5.3287 × 10^8^
	T_1_ → ^5^D_1_	5.2793 × 10^6^	^5^D_1_ → T_1_	5.5239 × 10^−3^
	T_1_ → ^5^D_0_	7.9416 × 10^6^	^5^D_0_ → T_1_	3.4140 × 10^−7^
[Eu_2_(Ibf)_6_(4,4′‐dMeObpy)_2_]	S_1_ → ^5^D_4_	1.4087 × 10^1^	^5^D_4_ → S_1_	1.1473 × 10^−14^
	T_1_ → ^5^D_1_	2.3707 × 10^6^	^5^D_1_ → T_1_	3.1690 × 10^−1^
	T_1_ → ^5^D_0_	4.7162 × 10^6^	^5^D_0_ → T_1_	2.5902 × 10^−5^
[Eu_2_(Ibf)_6_(5,5′‐dMeObpy)_2_]	S_1_ → ^5^D_4_	1.8843 × 10^3^	^5^D_4_ → S_1_	9.6860 × 10^−9^
	T_1_ → ^5^D_1_	9.8363 × 10^4^	^5^D_1_ → T_1_	9.6977 × 10^−5^
	T_1_ → ^5^D_0_	1.4746 × 10^5^	^5^D_0_ → T_1_	5.9731 × 10^−9^
[Eu_2_(Ibf)_6_(4,4′‐dhbpy)_2_]	S_1_ → ^5^D_4_	1.1140 × 10^2^	^5^D_4_ → S_1_	3.7718 × 10^−13^
	T_1_ → ^5^D_1_	6.3805 × 10^7^	^5^D_1_ → T_1_	5.5635 × 10^−1^
	T_1_ → ^5^D_0_	1.0846 × 10^8^	^5^D_0_ → T_1_	3.8854 × 10^−5^
[Eu_2_(Ibf)_6_(5,5′‐dhbpy)_2_]	S_1_ → ^5^D_4_	6.1013 × 10^3^	^5^D_4_ → S_1_	3.5256 × 10^−8^
	T_1_ → ^5^D_1_	1.0554 × 10^5^	^5^D_1_ → T_1_	2.7440 × 10^−4^
	T_1_ → ^5^D_0_	1.6730 × 10^5^	^5^D_0_ → T_1_	1.7872 × 10^−8^

*Note:* The evaluated forward channels include the singlet (S_1_ → ^5^D_4_) and triplet (T_1_ → ^5^D_1_, T_1_ → ^5^D_0_) transitions, alongside their respective reverse pathways (^5^D_4_ → S_1_, ^5^D_1_ → T_1_, ^5^D_0_ → T_1_), as determined by the LUMPAC software.

In general, the calculated back‐transfer rates are several orders of magnitude lower than the corresponding forward transfer rates, ensuring that the ^5^D_0_ emitting level is efficiently populated. However, two notable exceptions can be identified. The first involves the [Eu_2_(Ibf)_6_(4,4′‐dnbpy)_2_] complex, which simultaneously exhibits the lowest *W*_ET_ values for both the T_1_ → ^5^D_1_ (9.71 × 10^3^ s^−1^) and T_1_ → ^5^D_0_ (1.45 × 10^4^ s^−1^) channels across the series, alongside the highest *W*_BT_ rate for the ^5^D_4_ → S_1_ back‐transfer pathway (6.02 × 10^5^ s^−1^). This kinetic behavior is physically consistent with the exceptionally low singlet energy of the 4,4′‐dnbpy ligand (S_1_ = 25,176 cm^−1^, Table 9). Under these energetic conditions, the S_1_ → ^5^D_4_ transfer becomes thermodynamically reversible, and once the energy reaches the ^5^D_4_ state, it is efficiently transferred back to S_1_ due to the dominant *W*_BT_ (^5^D_4_ → S_1_) rate. Consequently, only a minor fraction of the absorbed photons successfully populates the ^5^D_0_ state via the triplet pathway. This photophysical bottleneck elucidates the anomalously low sensitization efficiency (19.45%) and quantum yield (17.61%) reported for this complex in Table 11. This case clearly illustrates how the energetic positioning of the S_1_ state can critically dictate the overall sensitization efficiency.

**TABLE 11:** Predicted total emission quantum yield (*Φ*) and sensitization efficiency ($\eta_{\text{sens}}$) calculated for the eight newly proposed systems, obtained from the excited‐state properties of the ligands and the ligand → Eu^3+^ energy transfer rates.

Complex	Quantum yield (%)	Sensitization efficiency (%)
[Eu_2_(Ibf)_6_(4,4′‐dabpy)_2_]	74.77	97.13
[Eu_2_(Ibf)_6_(5,5′‐dabpy)_2_]	77.33	87.20
[Eu_2_(Ibf)_6_(4,4′‐dnbpy)_2_]	17.61	19.45
[Eu_2_(Ibf)_6_(5,5′‐dnbpy)_2_]	62.77	98.29
[Eu_2_(Ibf)_6_(4,4′‐dMeObpy)_2_]	93.77	97.63
[Eu_2_(Ibf)_6_(5,5′‐dMeObpy)_2_]	66.88	70.38
[Eu_2_(Ibf)_6_(4,4′‐dhbpy)_2_]	79.06	98.95
[Eu_2_(Ibf)_6_(5,5′‐dhbpy)_2_]	54.74	72.46

The second exception is the [Eu_2_(Ibf)_6_(5,5′‐dnbpy)_2_] complex. Despite exhibiting an exceptionally high *W*_BT_(^5^D_4_ → S_1_) (5.33 × 10^8^ s^−1^, the highest in the series), it achieves an outstanding sensitization efficiency of 98.29% and a quantum yield of 62.77%. This apparent contradiction, especially when compared to its 4,4′‐isomer, is resolved by the remarkably *W*_ET_ for the triplet‐mediated pathways: *W*_ET_(T_1_ → ^5^D_0_) = 7.94 × 10^6^ s^−1^ and *W*_ET_(T_1_ → ^5^D_1_) = 5.28 × 10^6^ s^−1^. These elevated rates arise from the favorable combination of a relatively short energy donor–acceptor distance for the triplet state (*R*_L_ = 6.54 Å, Table 9) and a highly efficient intersystem crossing. The latter is driven by the narrow singlet‐triplet energy gap (1666 cm^−1^) characteristic of the electron‐withdrawing –NO_2_ group at the 5,5′‐position. Consequently, even with rapid back‐transfer from ^5^D_4_ to S_1_, the T_1_ → ^5^D_0_ pathway remains kinetically competitive and dominates the sensitization dynamics. Ultimately, the quantum yield of this complex (62.77%) nearly reaches its theoretical ceiling established by its intrinsic quantum efficiency (η = 63.85%, Table 8), aligning with its near‐unity sensitization efficiency (98.29%, Table 11). This kinetic profile conclusively demonstrates that the overall performance of this system is not limited by the sensitization step, but rather by the intrinsic radiative–nonradiative balance at the ^5^D_0_ level, inherently capped by its high nonradiative decay rate ($A_{\mathrm{NR}}$ = 276.89 s^−1^).

A comparative analysis of the [Eu_2_(Ibf)_6_(4,4′‐dMeObpy)_2_] and [Eu_2_(Ibf)_6_(5,5′‐dMeObpy)_2_] isomers reveals a pronounced regiochemical effect on the energy transfer kinetics. The 4,4′‐isomer exhibits *W*_ET_(T_1_ → ^5^D_0_) = 4.72 × 10^6^ s^−1^, which is approximately 32 times higher than that of its 5,5′‐isomer (1.47 × 10^5^ s^−1^), despite both systems possessing nearly identical *R*_L_ values for the triplet state (4.077 and 4.045 Å, respectively, Table 9). Given that *R*_L_ is essentially the same, this kinetic contrast is dictated by the energy gap between T_1_ and the ^5^D_0_ acceptor level. The T_1_ level of the 5,5′‐dMeObpy ligand (22,857 cm^−1^) is the highest in the studied series, lying approximately 5457 cm^−1^ above ^5^D_0_ (~17,400 cm^−1^). Within the framework of the Dexter exchange mechanism, the transfer rate decays approximately exponentially as the energy mismatch between the triplet level and the Eu^3+^ excited state increases. Consequently, the larger gap in the 5,5′‐isomer severely dampens its forward energy transfer rate. This mechanistic interpretation is consistent with the sensitization efficiencies of 97.63% (4,4′‐) versus 70.38% (5,5′‐), and their corresponding quantum yields (93.77% and 66.88%, Table 11). Ultimately, this highlights a fundamental rule of lanthanide sensitization: the T_1_ energy must strike a delicate balance, being neither too close to (risking back‐transfer) nor excessively far above (minimizing forward transfer) the ^5^D_0_ emitting level.

The [Eu_2_(Ibf)_6_(4,4′‐dhbpy)_2_] complex stands out by exhibiting the highest *W*_ET_ values in the series, specifically 6.38 × 10^7^ s^−1^ for the T_1_ → ^5^D_1_ channel and 1.08 × 10^8^ s^−1^ for T_1_ → ^5^D_0_. These rates are approximately one order of magnitude higher than those observed for the best‐performing dMeObpy isomers, directly driven by the near‐unity sensitization efficiency (98.95%) achieved by this complex. Nevertheless, despite achieving the highest sensitization efficiency alongside a high intrinsic quantum efficiency (η = 79.92%, Table 8), its overall quantum yield (79.06%, Table 11) remains bottlenecked by a relatively high nonradiative decay rate ($A_{\mathrm{NR}}$ = 104.36 s^−1^). This finding definitively confirms that maximizing the emission efficiency of these complexes requires the simultaneous optimization of the ligand‐to‐metal sensitization pathways and the suppression of nonradiative losses.

A comprehensive analysis of the kinetic data in Table 10 indicates that the overall emission quantum yield of the Eu^3+^ complexes in this series is governed by three sequential and competing factors: (i) the efficiency of ligand–metal energy transfer; (ii) the magnitude of energy back‐transfer; and (iii) the intrinsic radiative–nonradiative balance at the ^5^D_0_ level, which is dictated by the Judd–Ofelt parameters and the vibrational environment. The [Eu_2_(Ibf)_6_(4,4′‐dMeObpy)_2_] complex achieves the optimal balance among these factors. By combining an efficient T_1_ → ^5^D_0_ transfer (*W*_ET_ = 4.72 × 10^6^ s^−1^), negligible back‐transfer processes, and the lowest nonradiative decay rate in the entire series ($A_{\mathrm{NR}}$ = 17.76 s^−1^), this system delivers the highest quantum yield (93.77%).

An analysis of the calculated data reveals significant variations, with *R*_L_ ranging from 3.87 Å (for the triplet state of the 5,5′‐dabpy system) to 8.29 Å (for the singlet state of the 5,5′‐dnbpy system). Notably, the [Eu_2_(Ibf)_6_(5,5′‐dabpy)_2_] complex exhibits the shortest triplet *R*_L_ (3.87 Å), indicating that functionalization at the 5,5′ position places the ligand chromophore closer to the Eu^3+^ ion. This spatial proximity favors the short‐range Dexter mechanism, theoretically maximizing the sensitization process. This geometric advantage contextualizes the data in Table 11, where this complex achieves a sensitization efficiency of 87.20%. Interestingly, while the sensitization efficiency of the 5,5′‐dabpy complex is lower than that of its 4,4′‐isomer (97.13%), its overall quantum yield (77.33%) marginally surpasses that of the 4,4′‐analog (74.77%). This apparent inversion implies that the substantially shorter triplet *R*_L_ distance in the 5,5′‐isomer compensates for the less efficient sensitization step, directly enhancing the quantum yield.

## 5 Conclusions

In this work, a theoretical protocol for the design of novel Eu^3+^‐based luminescent systems is presented, developed from experimental data of a previously synthesized and characterized precursor complex. The central strategy consisted of preserving the coordination polyhedron geometry of the reference system while introducing modifications in the outer organic portion of the ligands. This approach ensured the reliable transferability of the parameters from the developed models to the newly proposed systems. To operate this protocol, two novel semiempirical models were developed.

The first model, based on the combination of the *QDC* and BOM approaches, enables the purely theoretical calculation of the Judd–Ofelt intensity parameters Ω_2_ and Ω_4_. Following parameterization using the [Eu_2_(Ibf)_6_(bpy)_2_] complex, the model was successfully validated to the analogous [Eu_2_(Ibf)_6_(4,4′‐dmbpy)_2_] and [Eu_2_(Ibf)_6_(5,5′‐dmbpy)_2_] complexes, yielding mean errors of only 1.7% and 3.9% for the Ω_2_ and Ω_4_ parameters, respectively.

The second model, based on the van Dijk–Schuurmans equation and derived via a Taylor series expansion, allowed the calculation of nonradiative decay rates with errors below 0.2% for the three studied systems. The integration of these two models permitted, for the first time in this class of compounds, the fully theoretical determination of radiative and nonradiative rates, the ^5^D_0_ excited‐state lifetime, and the intrinsic quantum efficiency, all in excellent agreement with experimental values.

The validated protocol was subsequently applied to the design of eight novel binuclear Eu^3+^ complexes by replacing the methyl groups in bipyridine with –NH_2_, –NO_2_, –OCH_3_, and –OH substituents at the 4,4′‐and 5,5′‐positions. An analysis of the Judd–Ofelt parameters showed that Ω_2_ values are strongly modulated by both the electronic nature and position of the substituents. Notably, the –NO_2_ group at the 5,5′‐position yielded the highest Ω_2_ value in the series (10.18 × 10^−20^ cm^2^), a result consistent with its strong electron‐withdrawing character and consequent asymmetry induced in the ligand field. In contrast, complexes bearing electron‐donating groups at the 4,4′‐positions, particularly the –OCH_3_ group, exhibited the lowest nonradiative rates. This behavior is attributed to the increased distance of C–H and O–H oscillators from the metal center in this substitution pattern, alongside the elimination of the deactivation pathway associated with the high‐frequency O–H oscillator (~3600 cm^−1^). As a result, the [Eu_2_(Ibf)_6_(4,4′‐dMeObpy)_2_] and [Eu_2_(Ibf)_6_(5,5′‐dMeObpy)_2_] complexes displayed the highest intrinsic quantum efficiencies in the series (η > 94%), achieving overall quantum yields of 93.8% and 66.9%, respectively. The difference between the two isomers is attributed to the superior sensitization efficiency of the 4,4′‐isomer (97.6% vs. 70.4%), demonstrating that the substituent position plays a decisive role not only in mitigating nonradiative decay but also in optimizing the ligand‐Eu^3+^ energy transfer. These theoretically predicted results identify [Eu_2_(ibf)_6_(4,4′‐dMeObpy)_2_] as the most promising candidate for the synthesis and testing of its highly efficient light‐emitting devices and luminescent sensors.

Finally, it is imperative to acknowledge that the proposed protocol carries limitations inherent to its semiempirical nature. Its applicability is restricted to systems whose coordination polyhedra are geometrically homologous to the reference complex used for parameterization, an essential condition for extracting reliable Judd–Ofelt intensity parameters. The development of more generalized models, capable of operating independently of prior experimental data, remains a primary objective for our future research. Nevertheless, the findings presented herein demonstrate that this computational approach represents a concrete and computationally accessible step toward the rational design of luminescent materials based on lanthanide ions.

## Funding

This work was supported by Conselho Nacional de Desenvolvimento Científico e Tecnológico (306015/2022‐6), Coordenação de Aperfeiçoamento de Pessoal de Nível Superior (FinanceCode001), Fundação de Amparo à Ciência e Tecnologia do Estado de Pernambuco (APQ‐0549‐1.06/17), and Fundação Carlos Chagas Filho de Amparo à Pesquisa do Estado do Rio de Janeiro (E‐26/200.863/2026).

## Conflicts of Interest

The authors declare no conflicts of interest.

## Data Availability

The data that support the findings of this study are available from the corresponding author upon reasonable request.
